# Pancreatic cancer cells infiltrate nerves through TGFbeta1-driven perineural epithelial-to-mesenchymal-like transdifferentiation

**DOI:** 10.1016/j.neo.2025.101126

**Published:** 2025-01-21

**Authors:** Theresa Krauss, Ibrahim Halil Gürcinar, Ulrike Bourquain, Maren Hieber, Evelyn N. Krohmer, Nan Wu, Sergey Tokalov, Rüdiger Goess, Carmen Mota Reyes, Dieter Saur, Helmut Friess, Güralp O. Ceyhan, Ihsan Ekin Demir, Okan Safak

**Affiliations:** a Department of Surgery, Klinikum rechts der Isar, Technical University of Munich, School of Medicine, Munich, Germany; b Institute of Translational Cancer Research and Experimental Cancer Therapy, TranslaTUM, Munich, Germany; c Department of Neurology and Neurophysiology, Medical Center - University of Freiburg, Faculty of Medicine, University of Freiburg, Freiburg, Germany; d Division of HPB Surgery, Acibadem Mehmet Ali Aydinlar University School of Medicine, Istanbul, Turkey; e German Cancer Consortium (DKTK), Munich site, Germany; f SFB 1321, Modelling and Targeting Pancreatic Cancer, Munich, Germany; g Else Kröner Clinician Scientist Professor for Translational Pancreatic Surgery, Germany; h Neural Influences in Cancer (NIC) Research Consortium, Germany; i Comprehensive Cancer Center München, Institute for Tumor Metabolism, TUM School of Medicine and Health, University Medical Center, Technical University of Munich, Germany

**Keywords:** Pancreatic cancer, TGFbeta1, Epithelial-to-mesenchymal-like transdifferentiation, Nerve infiltration

## Abstract

Neural invasion is a prognostic hallmark of pancreatic ductal adenocarcinoma (PDAC), yet the underlying mechanisms behind the disruption of perineural barriers and access of cancer cells into intrapancreatic nerves remain poorly understood. This study aimed to investigate the role of epithelial-mesenchymal transformation (EMT) in perineural epithelial cells during neural invasion.Histopathological analysis of human and murine primary tumors using perineurium-specific GLUT1 antibody revealed a reduction in perineural integrity, which positively correlated with the extent of neural invasion in human PDAC cases. Human pancreatic cancer cell lines were found to secrete TGFbeta1, which induced EMT of perineural epithelial cells, characterized by the loss of epithelial markers (CK19-9) and the acquisition of mesenchymal markers (alphaSMA, N-Cadherin). Additionally, these transitioning perineural epithelial cells demonstrated increased matrix-degrading capabilities through the upregulation of matrix-metalloproteases 3 and 9 via SMAD2. In an autochthonous mouse model with elevated endogenous TGFbeta1 levels in addition to oncogenic Kras activation (*Ptf1a^Cre/+^, LSL-Kras^G12D/+^, LSL-R26^Tgfβ/+^*), decreased perineural integrity could be reproduced *in vivo*.Collectively, these findings underscore the role played by TGFbeta1-overexpressing pancreatic cancer cells in the dismantling of perineural barriers during neural invasion.

## Highlights

• This study aimed to investigate the molecular mechanisms that lead to the loss of the perineural barrier during neural invasion in PDAC.
• Histopathological analysis of human and murine primary tumors revealed a decline in perineural integrity, which exhibited a positive correlation with the degree of neural invasion observed in human PDAC cases.
• Human pancreatic cancer cell lines were observed to secrete TGFbeta1, triggering mesenchymal-like transformations in perineural epithelial cells. This transformation was characterized by the acquisition of mesenchymal markers (alphaSMA, N-Cadherin) in perineural epithelial cells.
• Transitioning perineural epithelial cells exhibited heightened matrix-degrading capabilities due to the upregulation of matrix metalloproteases 3 and 9 via SMAD2.
• In an autochthonous PDAC mouse model featuring elevated endogenous TGFbeta1 levels alongside oncogenic Kras activation (*Ptf1a^Cre/+^, LSL-Kras^G12D/+^, LSL-R26^Tgfβ/+^*), a decrease in perineural integrity was observed.

## Graphical abstract

**Figure:**
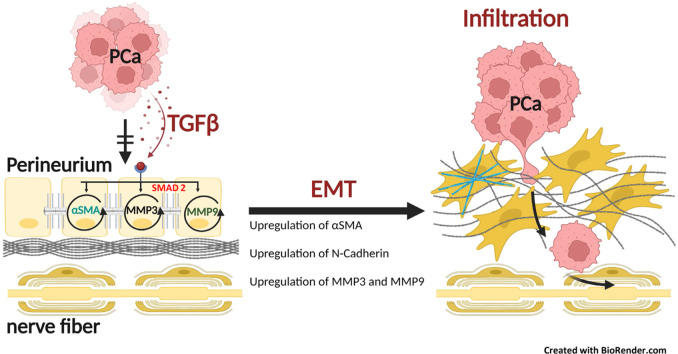

To infiltrate intrapancreatic nerves, pancreatic cancer cells (PDAC) secrete TGFbeta, inducing epithelial-mesenchymal-transformation (EMT) in perineural epithelial cells. Mesenchymal markers, such as AlphaSMA and N-Cadherin, are consequently upregulated in perineural epithelial cells. Furthermore, matrix-degrading capabilities of perineural epithelial cells are enhanced through the upregulation of MMP3 and MMP9 via SMAD2.

## Introduction

Despite significant advancements in scientific research over recent years, pancreatic cancer remains one of the deadliest forms of cancer, currently ranking as the fourth leading cause of cancer-related deaths in the United States [1]. A key factor contributing to the poor prognosis associated with pancreatic ductal adenocarcinoma (PDAC) is its aggressive invasive nature, particularly evident in the process of neural invasion (NI) [2]. In this process, PDAC cells actively penetrate both the epi-neural and peri-neural barriers, accessing a protected niche. This environment not only shields them from immune response, but also facilitates their uncontrolled spread towards the extrapancreatic nerve plexus [3]. In response to this invasion, neurons increase neurite outgrowth, creating a reciprocal relationship that intensifies the cancer's growth and spread, driven in part by neurotrophic factors like nerve growth factor (NGF) [4,5]. Although recent scientific investigations have increasingly focused on neural-epithelial interactions, the specific mechanisms that facilitate the disruption of the perineural barrier, thereby allowing cancer cells direct access into intrapancreatic nerves, remain largely unclear.

Peripheral nerves are sheathed by a tri-layered protective cover comprising the epineurium, the perineurium, and the endoneurium. The perineurium plays a crucial role in maintaining neural integrity and homeostasis. Composed of flattened epithelial cells arranged concentrically around each nerve fascicle and supported by a basement membrane, the perineurium forms a selective blood-nerve barrier through tight junctions in its inner layers [6]. Notably, perineural epithelial cells derive from mesenchymal cells in the vicinity of developing nerves, not from neural crest cells [7]. This mesenchymal-epithelial transformation (MET) during neurogenesis [8], and its counterpart, i.e., epithelial-mesenchymal transformation (EMT), are dynamic processes allowing cells to adjust their differentiation state under various conditions, including wound healing, embryogenesis, and tumor progression [[9], [10], [11]]. EMT, a multi-step 'fundamental reprogramming' of cell biology, entails the loss of epithelial characteristics and the acquisition of a mesenchymal phenotype, marked by changes in cell markers (loss of E-cadherin and gain of Vimentin, N-cadherin, alphaSMA) and enhanced migratory and invasive capabilities [[11], [12], [13]]. In the tumor microenvironment of human PDAC, factors such as chronic inflammation and TGFbeta are known to initiate EMT in cancer cells [14,15]. However, the potential role of EMT in perineural epithelial cells during perineural invasion (PNI) in PDAC remains unexplored.

In this study, we investigated whether PCa cells can induce the transdifferentiation of perineural epithelial cells into myofibroblastic mesenchymal cells. We found that TGFbeta-secreting PCa cells compromise the perineural barrier and promote NI by initiating EMT in perineural cells.

## Materials and methods

### Patients and histological specimens

Pancreatic tissue samples for immunohistochemistry from patients with PDAC and chronic pancreatitis (CP) were obtained during surgical resection in our institution between 2007 and 2012. All carcinoma cases were classified as conventional PDAC; patients with special tumor types such as intraductal papillary-mucinous neoplasms or secondary tumors were excluded from the study. Normal pancreatic tissue (NP) was collected from patients with non-cancerous indications for surgical procedures. In each cohort, a total number of 20 patients was included. Clinical and histopathological data were obtained by detailed retrospective review of medical records and showed no significant difference for the respective parameters in compared groups (table). Sections used for final analysis contained a desired amount of 9 nerves or more. As a result, we analyzed 182 nerves for NP, 190 nerves for CP and 200 nerves for PCa. All patients were informed and consented to the collection of tissue samples for research purposes. The study was conducted with the approval of the Ethics Committee at the Technical University of Munich, Germany (Approval Nr. 2016-550-S-SR).

### Immunohistochemistry and quantitative analysis of perineural integrity

Resected specimens were fixed in 4 % formalin and cut stepwise into 3 µm sections. Immunohistochemical visualization of the perineurium was conducted with insulin-dependent-glucose-transporter-1 (GLUT1) antibody, a highly specific marker for perineural epithelial cells [16]. After deparaffinization and rehydration, the samples were subject to heat-mediated antigen retrieval and blocking of unspecific labeling with normal goat serum and 3 % hydrogen peroxide. GLUT1 antibody (Abcam ab652, Cambridge, United Kingdom) was diluted at 1:2000 and incubated overnight at 4 °C in a humid chamber. Detection of immunoreactivity was conducted with horseradish-peroxidase (HRP) and subsequent color reaction with HRP-substrate DAB. All sections were counterstained with hematoxylin for better visual distinction of cell nuclei.

Tissue expression of GLUT1 was quantitatively compared for patients with PDAC, CP and NP. The nerves of each section were photographed at high resolution and 20x magnification with a digital microscope (Keyence BioRevo BZ-9000, Neu-Isenburg, Germany). For subsequent analysis of the GLUT1-stained perineural segments as well as the total nerve circumference, we used the ImageJ software (version 1.44p, NIH, USA). The ratio of the GLUT1-positive perineurium to the total nerve circumference was termed perineural integrity. In order to correlate perineural integrity with the severity of perineural invasion or neuritis (neuro-inflammation), we made use of a previously established scoring system for each nerve: “0 = no NI or mere epineural association”, “1 = perineural invasion or peri-neuritis”, and “2 = endoneural invasion or endo-neuritis” [[17], [18], [19]].

### Immunofluorescence staining

Formalin-fixed paraffin-embedded (FFPE) tissue sections (2.5 µm) were deparaffinized in Roticlear (3 × 10 min) and rehydrated through a graded ethanol series. Antigen retrieval was conducted in 10 mM citrate buffer (pH 6.0) at 95 °C for 20 min, followed by cooling to room temperature and washing with PBST (0.1 % Tween 20 in PBS). Tissues were permeabilized with 0.1 % Triton X-100 for 10 min, then blocked with 5 % BSA in PBST for 1 h. Primary antibodies, anti-PGP (1:2000, Novus NB110-58872) and anti-PANCK (1:200, Invitrogen MA5-13203), were applied overnight at 4 °C in 1 % BSA. Sections were washed and incubated with Alexa Fluor 488 (PANCK) and Alexa Fluor 647 (PGP) secondary antibodies (1:200) for 1 h at room temperature in the dark. Nuclei were counterstained with DAPI (1:10,000) for 5 min, washed, and mounted with fluorescence-compatible medium (DAKO). Images were captured using a Zeiss confocal microscope with optimized channel settings.

### Cell lines and cultivation

All cell lines were cultivated under recommended conditions at 37 °C with 5 % CO_2_ in 75 cm^2^ cell culture flasks in their respective media containing 10 % fetal bovine serum (FBS) and 1 % penicillin/streptomycin (PS). All human pancreatic tumor cell lines (Capan1, Panc1, Su86.86 and T3M4) were obtained from ATCC or were a kind gift by Dr. Metzgar (Duke University, Durham NC) and cultivated in RPMI 1640 (Sigma Aldrich, Missouri, USA). Human perineural epithelial cells, also termed human perineural fibroblasts, were obtained from ScienCell (Carlsbad, CA, USA) and cultivated in the ready-to-use human perineural epithelial cell medium provided by the company ScienCell (Carlsbad, CA, USA).

### ELISA – quantitative analysis of TGFbeta expression in human PCa cell lines

To quantify the TGFbeta secretion of different human PDAC cell lines (Panc1, SU86.86, T3M4), we performed the Quantikine ELISA (R&D Systems, Minneapolis, USA) for TGFbeta1 according to the manufacturer's instructions. The levels of MMP-3 and MMP-9 in the supernatants of HPNEC were quantified using Proteintech-KE00160 Human Total MMP-3 ELISA Kit and Proteintech-KE00164 Human MMP-9 ELISA Kit, respectively, according to the manufacturer's instructions.

### Western Blot – quantitative analysis of protein expression of PDAC-conditioned human perineural epithelial cells

For the collection of cell culture supernatants, human PDAC cell lines (Capan1, Panc1, Su86.86 and T3M4) were cultured to 80 % confluency, switched to FBS-free RMPI medium and incubated for 24 h. Human perineural epithelial cells were conditioned with tumor cell supernatants at a final concentration of 300 µg/ml for 96 h. The positive control contained recombinant human TGFbeta1 (R&D Systems, Minneapolis, USA) at a concentration of 50 µg/ml and the negative control consisted of FBS-free human perineural epithelial cells with and without RPMI. After cultivation, the cells were homogenized using 10 mM Tris-HCl buffer (pH 7.0) containing 1 mM EDTA and 20 mM KCl. Soluble fractions were processed with protease inhibitors and amounts of 50 µg protein were subjected to 10 % sodium dodecyl sulfate polyacrylamide gel electrophoresis (SDS-PAGE) under reducing conditions. Subsequent to the transfer of proteins to a polyvinylidene difluoride (PVDF) membrane, the samples were incubated with an array of antibodies (anti-CK19, anti-N-Cadherin, anti-alphaSMA, anti-Vimentin, anti-GAPDH). In a final step, the labeled proteins were visualized with peroxidase-labeled anti-mouse and anti-rabbit secondary antibodies. Each cell line was tested in three biological replicates.

### Western Blot – quantitative analysis of protein expression of TGFbeta1-conditioned human perineural epithelial cells

Human Perineural Cells (HPNC) at 70–80 % confluence were treated with TGF-β1 (10 ng/mL; R&D Systems, 7754-BH-025/CF) for 24 h. Post-treatment, cells were lysed with RIPA buffer containing protease and phosphatase inhibitors. Protein concentrations were determined using the BCA assay, and 30 µg of protein was resolved on an 8 % SDS-PAGE gel and transferred to a PVDF membrane. Membranes were blocked with 5 % milk in TBST for 1 h and incubated overnight at 4 °C with primary antibodies: SMAD4 (1:1000, CST 46535S), p-SMAD2 (S645/467, 1:1000, CST 3108S), and SMAD2/3 (1:1000, CST 8685S). HRP-conjugated secondary antibodies were applied for 1 h at room temperature. Protein bands were visualized using ECL substrate and imaged with the Bio-Rad ChemiDoc system. Band intensities were quantified using ImageJ and normalized to β-actin.

### RNA array study of PDAC - conditioned human perineural epithelial cells

To analyze the transcriptional profile of SU86.86-conditioned human perineural epithelial cells, we utilized the Human Epithelial to Mesenchymal Transition (EMT) RT² Profiler PCR Array. First, we extracted RNA from the conditioned human perineural epithelial cells lysates according to the manufacturer's instructions, followed by the transcription of RNA into cDNA using the RT^2^ First Strand Kit. Subsequently, the samples were mixed with the RT^2^SYBR Green / Fluorescein PCR master mix and pipetted in adequate amounts onto the PCR array plates. The signal was measured using Roche LightCycler® 480 system. For analysis, we referred to the online data analysis center of the manufacturer. Changes in protein expression were considered relevant if the expression was increased more than 1.5-fold.

### Matrigel invasion assay - conditioned human perineural epithelial cells

Human Perineural Epithelial Cells (HPNEC; ATCC CRL-4023) were cultured in Fibroblast Growth Medium (Innoprot, P60108) at 37 °C with 5 % CO₂ until 70–80 % confluency. Cells were trypsinized, counted, and prepared at 1 × 10⁵ cells/well. To induce epithelial-to-mesenchymal transition (EMT), cells were treated with 10 ng/mL TGF-β1 in serum-free medium for 24 h. For the Matrigel invasion assay, cells were seeded into Transwell inserts pre-coated with Matrigel, with 10 % FBS in the lower chamber as a chemoattractant. After 24 h at 37 °C, non-invading cells were removed, and invading cells were fixed, stained with crystal violet, and counted under a microscope. Invasion was quantified using ImageJ, and results were compared to untreated controls to assess TGF-β1-induced effects.

### Chromatin immunoprecipitation (ChIP) -PCR with conditioned human perineural epithelial cells

HPNEC at 70–80 % confluence were treated with TGF-β1 (10 ng/mL; R&D Systems, 7754-BH-025/CF) or solvent for 24 h. Chromatin immunoprecipitation (ChIP) was performed using the Imprint® ChIP Kit (Sigma-Aldrich, CHIP1-24RXN) following the manufacturer's protocol. Chromatin crosslinks were reversed at 65 °C for 4 h, and fragmented via sonication to 200–500 bp, verified by agarose gel electrophoresis. Fragmented chromatin was immunoprecipitated with anti-SMAD2 (1:100, CST 3108S), anti-SMAD 2/3 (1:25, CST 8685S), anti-SMAD4 (1:50, CST 46535S) or IgG control, captured on protein A/G magnetic beads, and eluted. DNA was purified using spin columns and analyzed by qPCR with SYBR Green Master Mix (Thermo Fisher) for MMP3 and MMP9 promoter regions. Primer sequences were:

MMP3: Fwd (5′-AACCTCACGCCAGTGACTTG-3′),

Rev (5′-AGGGCAGCAGTTGACAGGTT-3′)

MMP9: Fwd (5′-CCAGTAGACAACACGGACCA-3′),

Rev (5′-CACTTGTCGGCGATAAGGTA-3′) qPCR reactions (20 µL) included 10 ng input or ChIP DNA, 10 µL SYBR mix, and 0.5 µM primers, with cycling at 95 °C (10 min) followed by 40 cycles of 95 °C (15 s), 60 °C (30 s), and 72 °C (30 s). Relative enrichment was calculated using the ΔCt method, normalized to input DNA, and expressed as fold enrichment over IgG control.

### Genetically engineered mice with endogenous TGFbeta1-overexpression

In a final step, we assessed the perineural integrity of PDAC-specific TGFbeta1 overexpressing mice (*Ptf1a^Cre/+^, LSL-Kras^G12D/+^, LSL-R26^Tgfβ/+^*). After 12 weeks and subsequent tumor development, 7 mice were euthanized and subjected to histopathological analysis with GLUT1 antibody. 34 PK mice (*Ptf1a^Cre/+^, LSL-Kras^G12D/+^*) served as the control group. PK mice develop high grade PanIN lesions after 6 months [20]. Tissue processing and immunohistological staining was conducted according to the protocols for human specimens mentioned above. The mouse lines listed above will be published separately. The tissue collection from the primary tumor (pancreas) of the mice was performed at the time of sacrifice. The survival data of the mice is shown on Table 1.

**Table 1:** Genotypes and survival time of the mice.

mouse ID	genotype	survival (days)
P4269	p48-Cre +/-, LSL-Kras +/-	341
P3854	p48-Cre +/-, LSL-Kras +/-	274
P3922	p48-Cre +/-, LSL-Kras +/-	274
MZ1730	p48-Cre +/-, LSL-Kras +/-, LSL-Tgfß +/-	287
MZ1380	p48-Cre +/-, LSL-Kras +/-, LSL-Tgfß +/-	343
MZ1677	p48-Cre +/-, LSL-Kras +/-, LSL-Tgfß +/-	150

### Statistical analysis

All graphs are presented as mean ± SD. Two-group analyses were performed using the unpaired *t* test for continuous values and with the Mann–Whitney *u* test for scores and indices. Analyses of more than two groups were conducted using one-way ANOVA followed by Bonferroni's multiple comparison test. All tests were two-sided, and a *p* value < 0.05 was considered to indicate statistical significance.

## Results

### Perineural integrity is diminished in human pancreatic ductal adenocarcinoma (PDAC)

In this study, we employed the specific perineurium marker GLUT1 to evaluate perineural integrity within three distinct human pancreatic tissue types: Normal Pancreas (NP), Chronic Pancreatitis (CP), and Pancreatic Cancer (PDAC). These assessments were further correlated with specific histological features, including the extent of neural invasion and the incidence of pancreatic neuritis.

**Fig. 1:**
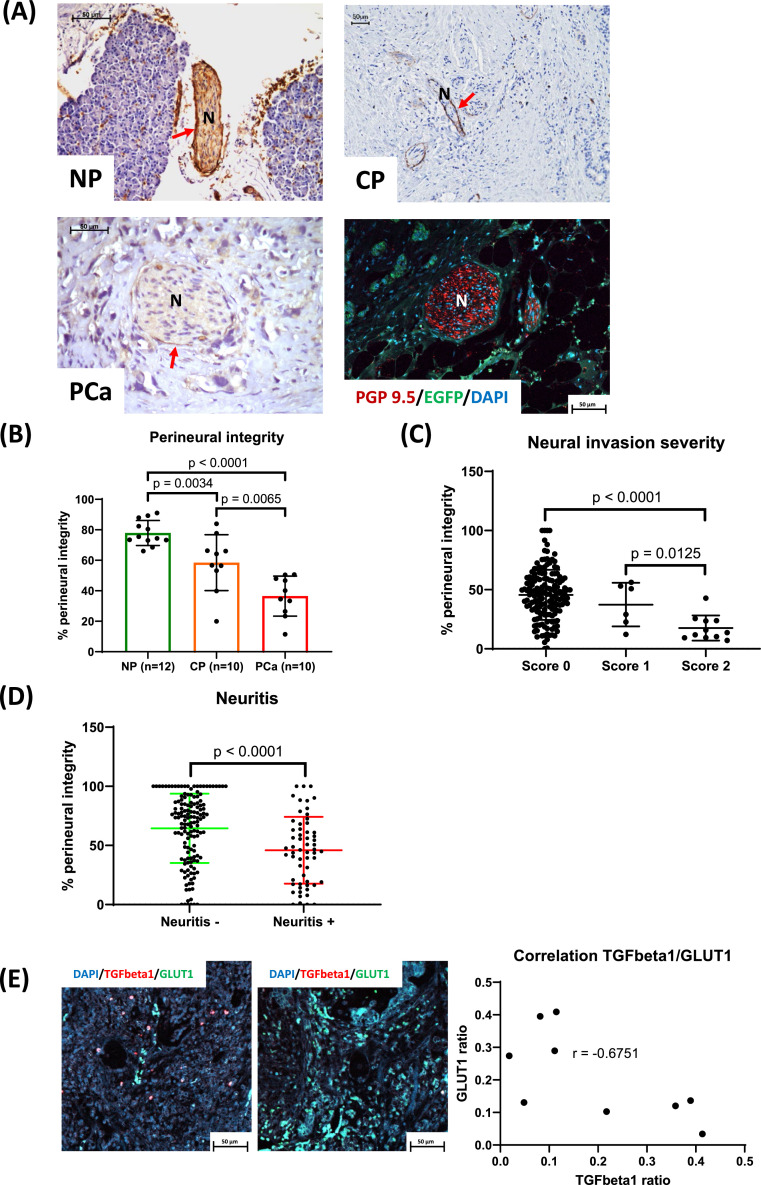
Perineural integrity in human pancreatic cancer (PDAC). **A.** Representative immunohistochemical photomicrographs (IHC). Nerve tissue sections from normal pancreas (NP), chronic pancreatitis (CP), and pancreatic cancer (PCa) were stained with anti-GLUT1 antibody. Representative immunofluorescent photomicrograph (IF). **B.** Perineural integrity was evaluated between the three different groups. **C.** Correlation between neural invasion score and perineural integrity. **D.** Relationship between neuritis and perineural integrity in CP. **E.** Representative immunofluorescent photomicrographs (IF). Graph shows correlation between TGFbeta1 and GLUT1; N, nerve; red arrows show the perineurium.

Insulin-dependent-glucose-transporter-1 (GLUT1) serves a critical role in facilitating glucose provision to neural tissues, primarily by mediating translocation across the blood-brain barrier [21]. Predominantly expressed in perineural epithelial cells (also termed perineural fibroblasts), GLUT1 is established as a highly specific marker, central to this study [16]. We found in NP pronounced immunoreactivity along the perineural circumference of intrapancreatic nerves, indicative of extensive GLUT1 expression (Fig. 1A). Moreover, inner nerve compartments also exhibited labeling, albeit at a lower degree, suggesting a potential GLUT1 presence in endoneurial blood vessels [22]. Notably, there was a marked disparity in perineurium staining intensity across the examined groups (CP, NP, PDAC; Fig. 1A). Quantitative analysis elucidated a significant reduction in perineural integrity within PDAC tissues compared to CP and NP (CP: 58.46 ± 18.35 % vs. PCa: 36.50 ± 13.14 %, *p* = 0.0065; NP: 77.95 ± 8.2 % vs. PCa: 36.50 ± 13.14 %, *p* < 0.0001; Fig. 1B). A clear correlation was observed in PDAC between perineural integrity and the neural invasion severity (NIS). Specifically, a neural invasion score of 2, denoting the infiltration of cancer cells into the endoneural space, correlated with a pronounced decrement in perineural integrity, in contrast to cases with an invasion score of 0 (NIS 2: 17.56 ± 10.63 vs. NIS 0: 45.92 ± 20.91, *p* < 0.0001; Fig. 1C). Besides, a reciprocal relationship was also found between neuritis in CP and perineural integrity, with specimens showing neuritis having significantly more perineural damage than those without inflammation (Neuritis+: 45.93 ± 28.24 vs. Neuritis-: 64.49 ± 29.31, *p* < 0.0001; Fig. 1D). In addition, the analysis of human carcinoma tissue sections revealed a significant negative correlation between TGFβ1 and GLUT1 densities within tumor cells and their associated nerve fibers (r = -0.6751; Fig. 1E).

These findings underscore the potential role of inflammatory and neoplastic processes in facilitating the degradation of protective perineural structures.

### TGFbeta1-hypersecreting PCa cells induce EMT-like changes in human perineural epithelial cells

**Fig. 2:**
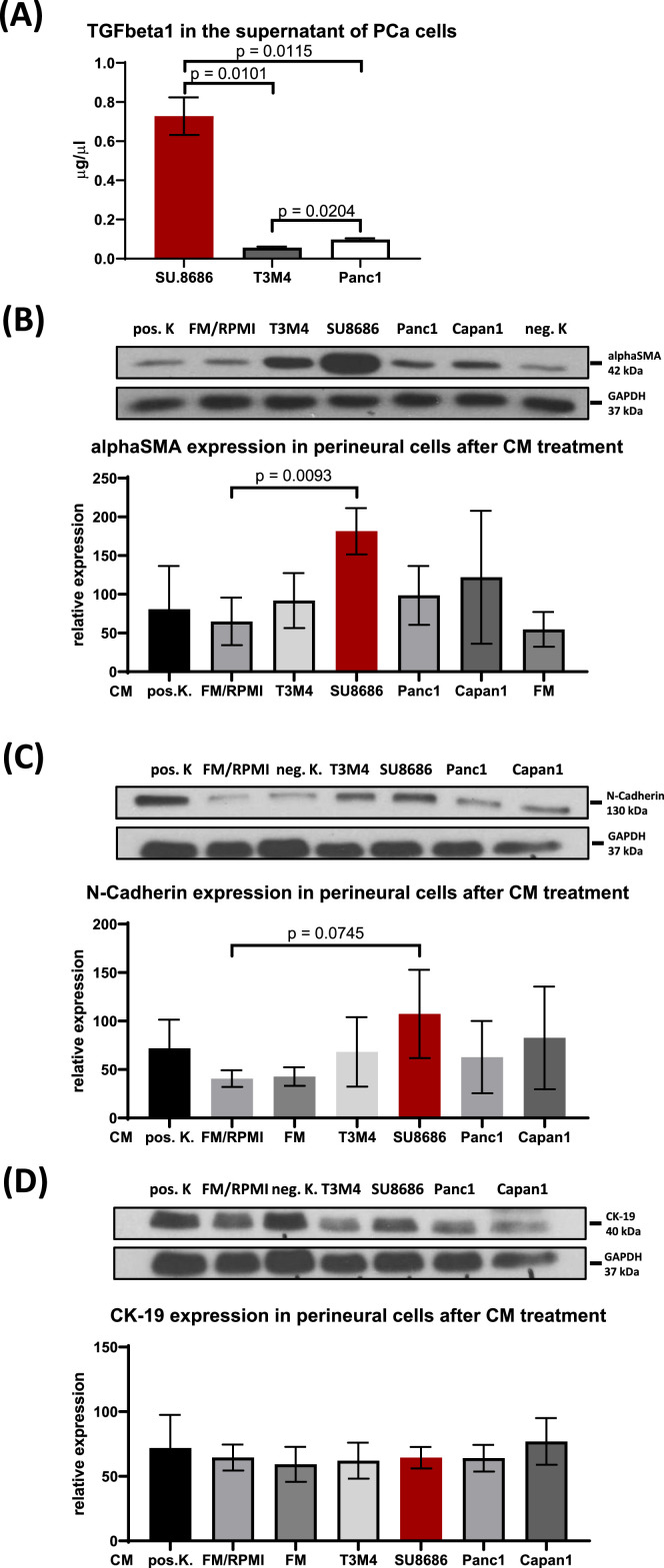
TGFbeta1-secretion and impact of tumor cell supernatants on the expression of alphaSMA, N-Cadherin and cytokeratin 19 (CK 19) in human perineural epithelial cells (HPNEC). **A.** Assessment of TGFbeta1-secretion in the supernatants of Panc1, SU86.86 and T3M4 via ELISA **B.** Representative western blot images of the alphaSMA content in perineural cells after conditioned media (CM) treatment. Graph representing the relative alphaSMA content in HPNEC after CM treatment with the supernatants of the different cancer cell lines. **C.** Representative western blot images of the N-Cadherin content in HPNEC after conditioned media (CM) treatment. Densitometry graph representing the relative N-Cadherin content of HPNEC after CM treatment with the supernatants of the different cancer cell lines. **D.** Representative western blot images of the CK19 content in HPNEC after conditioned media (CM) treatment. Densitometry graph representing the relative CK19 content of HPNEC after CM treatment with the supernatants of the different cancer cell lines.

In PDAC, EMT is widely spread in the tumor microenvironment and strongly affects tumor progression, owing to the abundance of EMT-promoting factors like TGFbeta1. We hypothesized that perineural epithelial cells might also undergo EMT in the TGFbeta1-rich microenvironment of PDAC, and that PDAC cells might be a major source of TGFbeta1. In our analysis, we quantitatively assessed TGFbeta1 secretion in the supernatant of various PDAC cell lines, including Panc1, SU86.86, and T3M4, using Quantikine ELISA. Known for inducing downstream ETM signaling in pancreatic cancer, TGFbeta is a critical focus in this context [14,23]. While all cell lines produced TGFbeta1, SU86.86 exhibited the highest expression (SU86.86: 0.728 ± 0.096 vs. Panc1: 0.098 ± 0.006 vs. T3M4: 0.056 ± 0.0057; Fig. 2A).

To investigate the impact of tumor cell supernatants on EMT in perineural epithelial cells, we conducted immunoblot analyses on cancer cell-conditioned human perineural epithelial cells. The analysis included various mesenchymal markers (alphaSMA, N-Cadherin) and an epithelial marker (CK 19-9) to evaluate different stages of cellular differentiation. An upregulation of mesenchymal markers coupled with a downregulation of epithelial markers was interpreted as EMT.

Compared to negative control, supernatants rich in TGFbeta1 (e.g., from the SU86.86 cell line) significantly increased the expression of mesenchymal markers such as alphaSMA (SU86.86: 181.5 ± 29.9 vs. FM/RPMI: 64.94 ± 30.77, *p* = 0.0093; Fig. 2B) and N-Cadherin (SU86.86: 107.3 ± 45.5 vs. FM/RPMI: 40.66 ± 8.53, *p* = 0.0745; Fig. 2C), while inducing no change in the epithelial marker CK 19-9 content of perineural epithelial cells (Fig. 2D). Conversely, cell lines with lower TGFbeta secretion, like T3M4 or Panc1, induced minor or no significant changes in the amounts of these markers in perineural epithelial cells. These results suggested that PDAC cells with high TGFbeta1 secretion can promote mesenchymal-like changes in perineural epithelial cells by activating latent EMT programs.

### Induction of MMP 3 and 9 upregulation by TGFbeta1-rich PDAC cell lines in human perineural epithelial cells

We utilized an RT^2^ profiler PCR array to analyze SU86.86-conditioned human perineural epithelial cells, focusing on the transcriptional alterations related to EMT signaling.

**Fig. 3:**
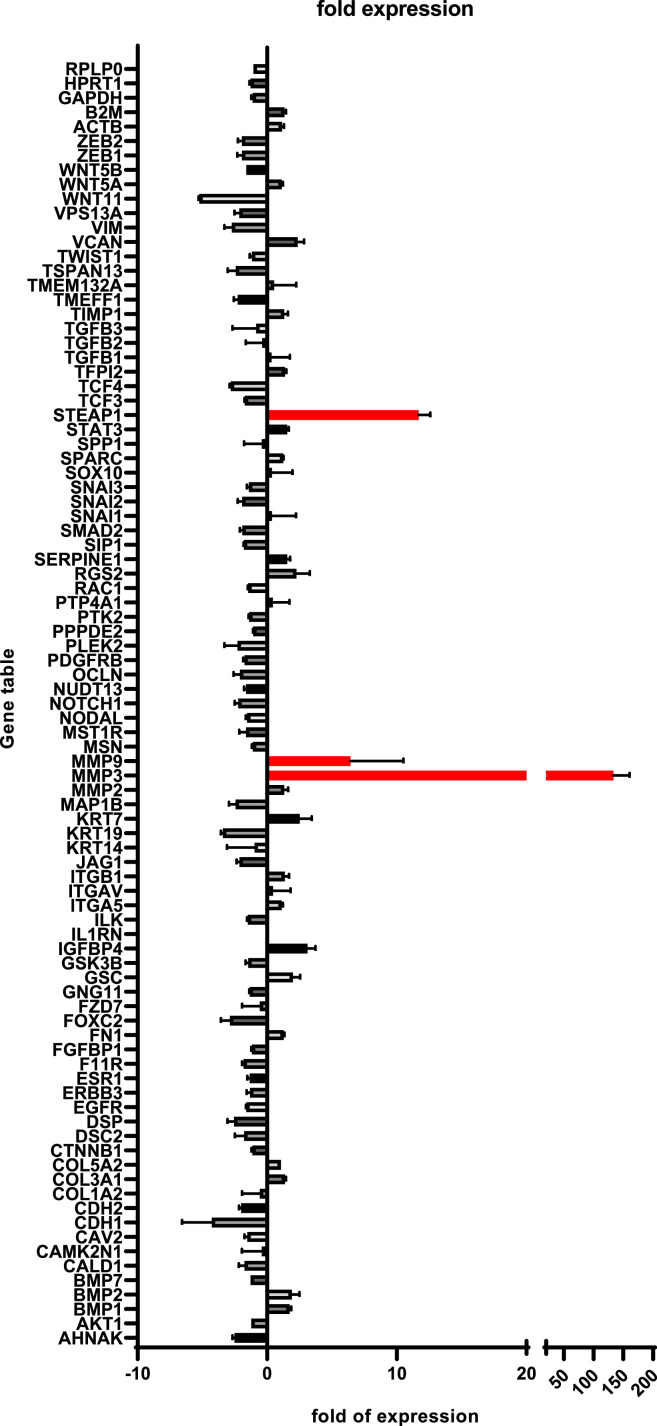
Transcriptional effects of SU86.86-conditioned media on human perineural epithelial cells Graph shows the gene expression profile of SU86.86-conditioned human perineural epithelial cells, as assessed with the he Human Epithelial to Mesenchymal Transition (EMT) RT² Profiler PCR Array. Significantly increased expression levels of upregulated genes are highlighted in red.

The analysis revealed that TGFbeta1-enriched tumor cell supernatants significantly augmented the expression of proteolytic enzymes in perineural epithelial cells. Notably, matrix metalloproteases (MMP) 3 and 9 were upregulated by 150- and 10-fold, respectively (Fig. 3). MMPs play a crucial role in tumor invasion and metastasis by degrading extracellular matrix components, facilitating cancer cell migration [24]. Additionally, a 13-fold increase in the transcription factor STEAP1 (Six Transmembrane Epithelial Antigen of the Prostate 1) was observed (Fig. 3) [[25], [26], [27]]. STEAP1 is overexpressed in several types of cancer, including prostate, bladder, and colon cancer, making it a potential biomarker for these diseases [28].

These results suggested that human perineural epithelial cells, upon transformation with elevated MMP 3 and 9, could acquire matrix-degrading capabilities, potentially aiding in the disruption of perineural barriers during neural invasion.

### Perineural sheet degradation is induced in HPNEC by elevation of MMP3 and MMP9 expression via SMAD2

**Fig. 4:**
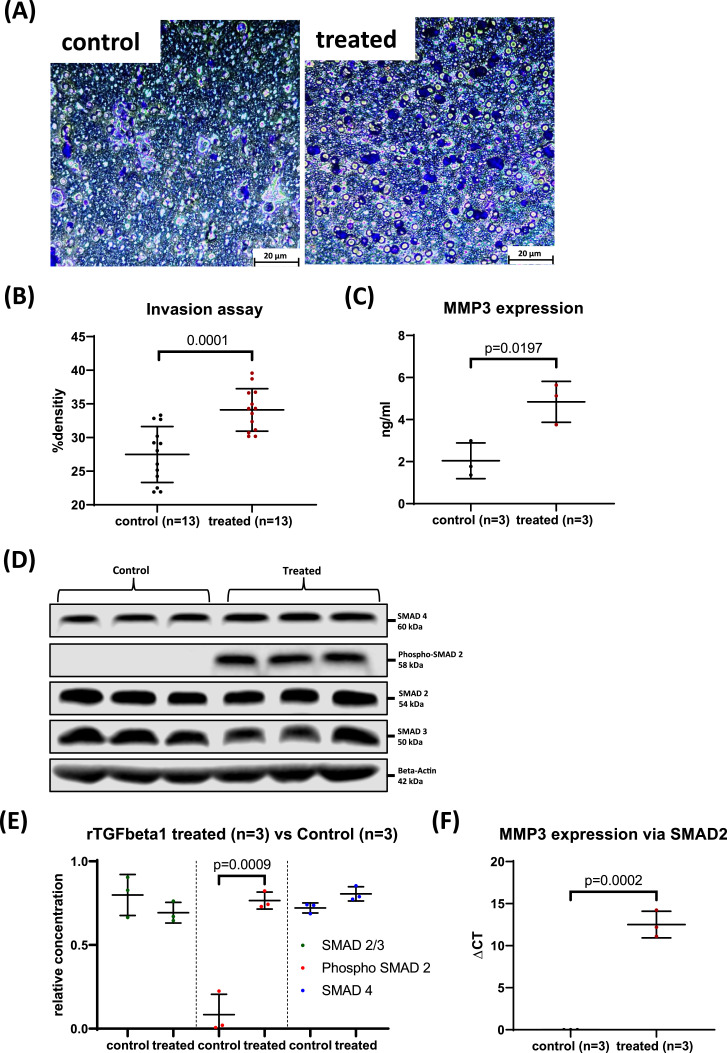
Matrigel Invasion Assay of TGFβ1-Treated HPNEC: Invasion density, MMP expression and transcriptional regulation **A.** Representative immunohistochemical photomicrographs (IHC) of Matrigel Invasion assay. Invading HPNEC are stained with crystal violet. **B.** Graph depicting the density of invading HPNECs after rTGFbeta1 treatment compared to untreated controls. **C.** Graph illustrating MMP3 content in the supernatant HPNEC following rTGFbeta1 treatment versus untreated controls. **D.** Representative western blot images showing SMAD protein content in human perineural cells post-TGFbeta1 treatment, highlighting the presence of phosphorylated SMAD2 exclusively in the treated group. **E.** Graph displaying the relative concentrations of various SMAD proteins in TGFbeta1-treated versus untreated HPNECs. **F.** Graph showing Chip-PCR results against MMP3 expression after immunoprecipitation against SMAD2 in TGFbeta1-treated HPNECs compared to control cells.

To reinforce our hypothesis that perineural sheath degradation is mediated by elevated expression of MMP3 and MMP9, we conducted Matrigel invasion assays on human perineural cells treated with TGFβ1 (10 ng/mL) for 30 min at 37 °C, comparing the results to untreated control groups (Fig. 4A). The assay demonstrated a prominent increase in the density of TGFβ1-treated perineural cells infiltrating the matrix compared to the controls (control: 27.48 ± 4.16 vs. treated: 34.11 ± 3.16, *p* = 0.0001; Fig. 4B). Furthermore, we quantified MMP3 and MMP9 content in the supernatant of the treated cells via ELISA. TGFβ1 treatment markedly increased MMP3 content (control: 2.04 ± 0.85 vs. treated: 4.84 ± 0.97, *p* = 0.0197; Fig. 4C), while an upward trend in MMP9 expression was observed (data not shown). Additionally, phosphorylated SMAD2 levels in the HPNEC were significantly elevated following TGFβ1 treatment (control: 0.08 ± 0.12 vs. treated: 0.77 ± 0.05, *p* = 0.0009; Fig. 4D, E).

To further elucidate the regulatory mechanism, chromatin immunoprecipitation (ChIP) PCR analysis revealed that TGFβ1 stimulation induced a robust increase in SMAD2-mediated MMP3 expression in human perineural cells (control: 0.00 ± 0.00 vs. treated: 12.51 ± 1.5, *p* = 0.0002; Fig. 4F).

Collectively, these findings underscore the pivotal role of TGFβ1-induced SMAD2 activation in driving MMP3 upregulation and facilitating perineural sheath degradation.

### Genetically engineered mice with endogenous TGFbeta1-overexpression exhibit reduced perineural integrity

**Fig. 5:**
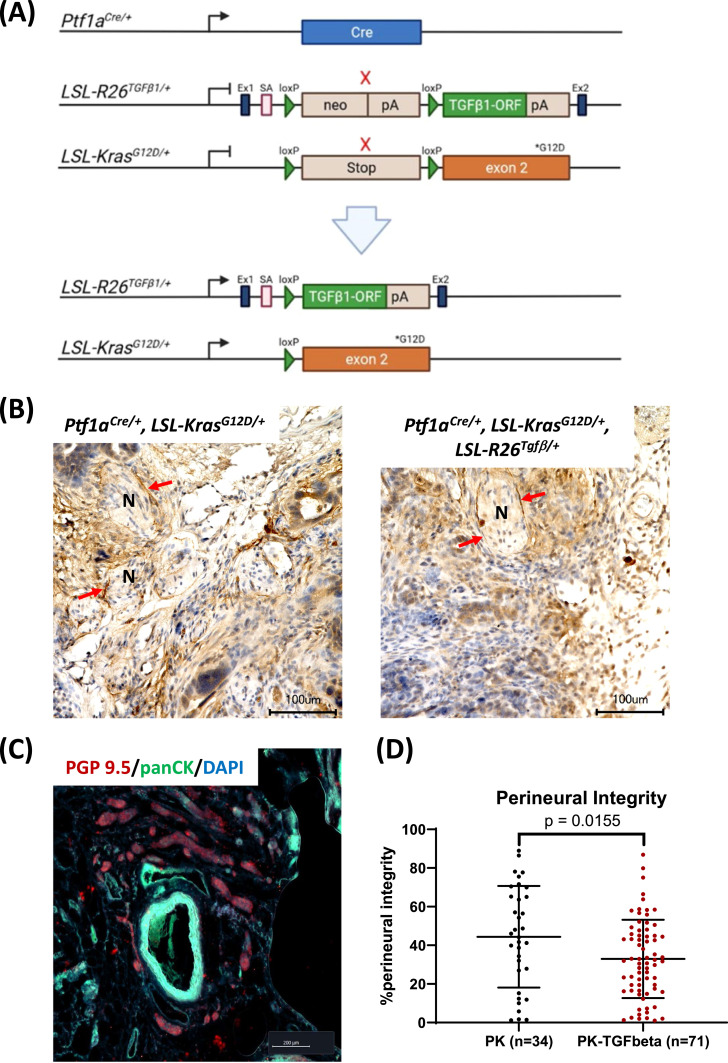
Comparison of the perineural integrity in a murine pancreatic cancer model with endogenous TGFbeta1 overexpression. **A.** Crossing scheme for breeding of PDAC cell-specific TGFbeta1 overexpression (*Ptf1a^Cre/+^, LSL-Kras^G12D/+^, LSL-R26^Tgfβ/+^*, https://mediatum.ub.tum.de/doc/1661773/document.pdf). **B.** Representative immunohistochemical photomicrographs (IHC). Nerve sections from PK (*Ptf1a^Cre/+^, LSL-Kras^G12D/+^*) mice and mice with endogenous TGFbeta1 overexpression (*Ptf1a^Cre/+^, LSL-Kras^G12D/+^, LSL-R26^Tgfβ/+^*) were stained with the GLUT1 antibody. **C.** Representative immunofluorescent photomicrograph (IF) of PGP9.5^+^ nerves from PK mice. Cancer cells were co-immunostained with antibodies against pan-cytokeratin (panCK). **D.** Graph shows the perineural integrity in TGFbeta1-overexpressing mice compared with regular PK mice (n = 3 mice, in brackets on the x-axis, the number of analyzed nerves is shown); N, nerve; red arrows show perineurium.

We then sought to validate the in vitro results through an in vivo approach, using a genetically engineered mouse model of PDAC with endogenous TGFbeta1 overexpression (*Ptf1a^Cre/+^, LSL-Kras^G12D/+^, LSL-R26^Tgfβ/+^*) (Fig. 5A). The immunohistochemical (Fig. 5B) and immunofluorescent examination (Fig. 5C) revealed that the perineural integrity in TGFbeta1-overexpressing mice was significantly compromised (32.95 ± 20.24 %) compared to the control group, which consisted of regular “PK” (*Ptf1a^Cre/+^, LSL-Kras^G12D/+^*) mice (44.41 ± 26.26 %, *p* = 0.0155; Fig. 5D).

## Discussion

Neural invasion is an independent prognostic factor for worse overall survival in PDAC, and a major trigger for local tumor recurrence and severe pain. Thus, understanding its mechanisms may have translational implications.

Our study revealed that PDAC cells trigger a "mesenchymal metamorphosis" in perineural epithelial cells, which grants cancer cells access into the interior of nerves. This process is largely driven by the secretion of TGFbeta1 by PDAC cells, both in vitro and in vivo. We also observed that human perineural epithelial cells, once transformed, begin to express increased levels of MMP 3 and 9. This upregulation via SMAD2 equips these cells with enhanced abilities to degrade the extracellular matrix. The correlation between high endogenous TGFbeta1 levels and a reduction in perineural integrity in vivo was found to be significant. Together, these findings provide strong evidence that TGFbeta1 plays a central role in the breakdown of the perineurium in PDAC. This is achieved through the activation of EMT programs in human perineural epithelial cells, leading to their transdifferentiation into mesenchymal-like cells. Ultimately, different tumor subtypes of human PDAC might have distinctive TGFbeta1-mediated effects on EMT signaling and perineural invasion.

The concept of EMT in perineural epithelial cells during neural invasion (NI) in PDAC is unexplored in current literature. In contrast, the transdifferentiation of PDAC cells into a mesenchymal state is a well-recognized phenomenon in PDAC [29,30]. This process often involves a 'cadherin switch', characterized by the downregulation of E-cadherin and upregulation of N-cadherin [13]. This switch is considered a key marker of cancer progression and metastasis, frequently correlating with poor clinical outcomes [29]. TGFbeta1 is acknowledged as a major driver of EMT signaling [23,31]. However, its role in cancer progression is complex and somewhat contradictory. In the early stages of tumor development, TGFbeta1 can act as a tumor suppressor, inducing growth arrest in cancer cells. In contrast, in later stages, it assumes the role of an oncogene, primarily due to its potent ability to initiate EMT programs. This dual functionality of TGFbeta1 reflects its varying influence depending on the cell cycle and tumor stage.

Nakajima et al. conducted a histopathological study demonstrating that N-cadherin expression in pancreatic cancer is stimulated by TGFbeta1. Furthermore, their research found a positive correlation between N-cadherin expression and the extent of neural invasion in pancreatic cancer [12]. Similar to Nakajima et al.’s findings, our study observed dynamic changes in adhesion molecules, particularly in relation to TGFbeta1. In perineural epithelial cells undergoing transition, TGFbeta1-enriched PDAC cell supernatants led to an increase in mesenchymal markers (such as alphaSMA and N-cadherin) and a decrease in epithelial markers (like CK 19-9). Consistent with the effects on adhesion molecule expression, high endogenous levels of TGFbeta1 in our study were also associated with reduced perineural integrity in vivo.

Our research contributes to the existing body of knowledge by establishing a novel connection. We identified that TGFbeta1-mediated EMT programs in perineural epithelial cells, which are part of the PDAC tumor microenvironment, play a significant role in neural invasion. This finding contrasts with previous studies that primarily focused on the role of TGFbeta1 in PDAC cells.

Several anti-TGFbeta agents and their therapeutic impact in PDAC are currently under clinical evaluation [32]. Trabedersen, an anti-TGFbeta2 agent, demonstrated effectiveness in mouse models and has progressed through phase I/II clinical trials, suggesting potential benefits in treating PDAC [33,34]. Galunisertib, an inhibitor of TGFbeta receptor type 1 (TGFbetaR1), has been noted for improving overall survival rates in patients with unresectable PDAC, especially when used in combination with Gemcitabine [35,36]. Despite these advancements, there remains a significant gap in data regarding the effectiveness of anti-TGFbeta1 therapies specifically targeting neural invasion in PDAC. Therefore, the specific impact of targeting TGFbeta1 on neural invasion and patient survival remains an area requiring further investigation.

In addition to TGFbeta, various downstream pathways are known to activate EMT programs in pancreatic cancer (PCa) [14]. Inflammatory signaling, in particular, has been previously identified as a significant promoter of EMT, as well as metastasis and invasion in cancer [15]. Our observations align with these findings, showing a positive correlation between pancreatic neuritis in patients with CP and the degree of NI in patients with PCa. Furthermore, the severity of perineural impairment was found to be associated with both conditions. This suggests that the prevalence of inflammatory processes in PDAC may not only contribute to but also hasten the degradation of perineural sheaths.

MMPs are critical in tumor progression and metastasis. Their role extends beyond digesting extracellular matrix (ECM) components. MMPs are involved in releasing cell-bound growth factors, activating other MMP precursors, and inhibiting MMP inhibitors [37]. These diverse functions make them key players in cancer biology. Recent studies, particularly in gastric cancer, have shown that MMPs are upregulated following the activation of EMT. This upregulation aids in facilitating tumor invasion and metastasis, underlining the interconnectedness of MMP activity and EMT processes [38]. Evidence from various researchers supports the idea that MMPs are not just involved in progressing EMT but may also initiate EMT programs. Radinsky et al. found that treatment of SCp2 mouse mammary epithelial cells with MMP 3 led to mesenchymal-like cell changes, including the downregulation of epithelial markers [24]. Similarly, Tan et al. reported that MMP 9 induces EMT in renal tubular cells, contributing to tubular fibrosis [39]. In our study, we clearly demonstrated that MMP3 and 9 are upregulated through EMT activation in perineural epithelial cells. This finding is pivotal in understanding the complex interplay between EMT and MMP activity in the context of cancer progression. The possibility that MMP3 and 9 could further enhance EMT transformation during neural invasion, potentially creating a cycle of mutual reinforcement, is an intriguing aspect that warrants further investigation.

SMAD2 is a pivotal mediator of TGF-β signaling, orchestrating processes such as cell proliferation, differentiation, extracellular matrix (ECM) remodeling and immune responses. Upon TGF-β stimulation, SMAD2 undergoes phosphorylation, forms a complex with SMAD4 and translocate to the nucleus to regulate gene expression, often in conjunction with other transcription factors [40]. In fibrotic diseases, SMAD2 activation promotes collagen production and ECM degradation through the induction of matrix metalloproteinases (MMPs) [41]. In cancer, SMAD2 exhibits dual roles: it can function as a tumor suppressor by inhibiting cell proliferation, and conversely, it can promote metastasis by facilitating epithelial-to-mesenchymal transition (EMT) [42].

Dysregulation of SMAD2 is implicated in various pathologies, including fibrosis and cancer, making it a potential target for therapeutic interventions and a biomarker for disease progression [43].

In conclusion, the present study has provided an insight into the mechanisms that facilitate cancer cell access into intrapancreatic nerves during neural invasion in PDAC. There seems to be a causal link between cancer cell-derived TGFbeta1-mediated EMT in perineural epithelial cells and the subsequent disruption of perineural barriers in cancer. These findings not only enhance our knowledge of cancer progression but also open new avenues for the treatment of neural invasion in PDAC.

## CRediT authorship contribution statement

**Theresa Krauss:** Writing – original draft, Visualization, Validation, Methodology, Investigation, Formal analysis, Data curation, Conceptualization. **Ibrahim Halil Gürcinar:** Data curation, Formal analysis, Investigation. **Ulrike Bourquain:** Validation, Methodology, Investigation, Formal analysis, Data curation. **Maren Hieber:** Methodology, Investigation, Conceptualization. **Evelyn N. Krohmer:** Writing – review & editing, Validation, Methodology, Investigation. **Nan Wu:** Investigation. **Sergey Tokalov:** Investigation. **Rüdiger Goess:** Writing – review & editing, Investigation. **Carmen Mota Reyes:** Validation, Investigation. **Dieter Saur:** Visualization, Supervision, Conceptualization. **Helmut Friess:** Validation, Supervision. **Güralp O. Ceyhan:** Validation, Supervision, Project administration. **Ihsan Ekin Demir:** Writing – review & editing, Validation, Supervision, Resources, Project administration, Methodology, Investigation, Conceptualization. **Okan Safak:** Writing – review & editing, Writing – original draft, Visualization, Validation, Software, Formal analysis, Data curation.

## Declaration of competing interest

The authors declare that they have no known competing financial interests or personal relationships that could have appeared to influence the work reported in this paper.
